# Antibody Discovery and Therapeutics in Spondyloarthritis: Recent Advances and Future Directions

**DOI:** 10.3390/antib15050090

**Published:** 2026-10-05

**Authors:** Kwai Wei Chan, Ghee Chong Koo, Che Kang Lim

**Affiliations:** 1 Department of Clinical Translational Research, Singapore General Hospital, Outram Rd, Singapore 169608, Singapore; 2 School of Biomedical Science and Pharmacy, Newcastle Australia Institute of Higher Education, 100 Victoria St, #13-01/02, Singapore 188064, Singapore; 3 Medicine Academic Clinical Programme, Duke-NUS Medical School, 8 College Rd, Singapore 169857, Singapore

**Keywords:** spondyloarthritis, inflammation, pathogenesis, antibody, DMARDs, cytokine, machine learning, artificial intelligence, therapeutics

## Abstract

Spondyloarthritis is a multi-faceted condition housing a group of inter-related chronic inflammatory rheumatic diseases such as ankylosing spondylitis, IBD-associated arthritis, psoriatic arthritis and juvenile idiopathic arthritis. While decades of research and technological advancements have significantly improved our understanding of the disease, there remain many underlying genetic and physiological mechanisms which are unsolved but essential for the development of the right diagnosis and treatment. This mini-review examines our current foundational knowledge of spondyloarthritis, the main concepts related to the disease pathogenesis and the various lines of therapies developed thus far. We hope to provide a perspective on the current and potential antibody discovery platforms for the next decade as computational methods and artificial intelligence become increasingly mature and relevant in clinical research.

## 1. Introduction

Spondyloarthritis (SpA) is not a single disease entity but rather an umbrella term encompassing a group of inter-related chronic inflammatory rheumatic diseases that share common clinical manifestations, genetic predispositions, and pathogenic mechanisms. These conditions are characterized primarily by inflammation of the axial skeleton, peripheral joints, and entheses, leading to chronic pain, stiffness as well as functional impairment. Persistent inflammation may further develop into aberrant bone formations such as syndesmophytes and ankylosis, which contribute to progressive loss of spinal mobility and disability.

**Figure 1:**
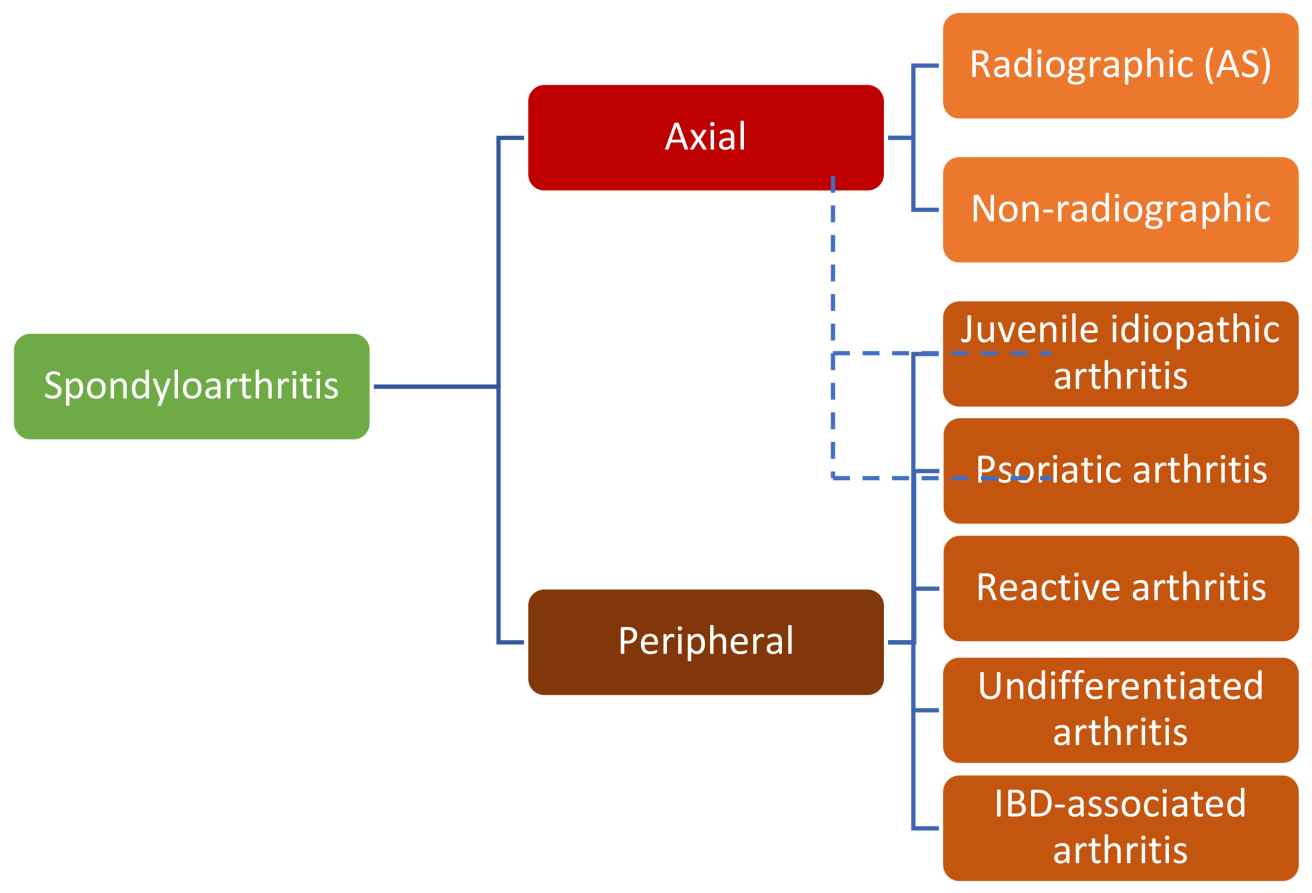
A hierarchical classification of the different subtypes of spondyloarthritis categorized mainly into axial and peripheral. JIA and PsA also have overlaps with axial manifestations.

SpA is broadly classified into two major categories: axial spondyloarthritis (axSpA), which predominantly affects the spine and sacroiliac joints, and peripheral spondyloarthritis (pSpA), which mainly involves the peripheral joints, entheses, and related structures (Figure 1). Despite being under the same disease family, symptoms can present broadly and heterogeneously in affected individuals. AxSpA, which can be further divided into two subtypes: radiographic (r-axSpA, also known as ankylosing spondylitis, AS) and non-radiographic (nr-axSpA), primarily manifests as inflammatory back pain [1] affecting the axial skeleton (spine and sacroiliac joints), though this is not a definitive symptom. In contrast, pSpA has five subtypes: reactive arthritis (RA), psoriatic arthritis (PsA), IBD-associated arthritis (also known as enteropathic arthritis, EA), undifferentiated spondyloarthritis (uSpA) and juvenile idiopathic arthritis (JIA). It primarily manifests in the peripheral joints (knees, fingers, toes), entheses, and even in tendons outside the spine, and is typically asymmetrical.

One of the most well-known features of spondyloarthritis (SpA) is its strong genetic association with the human leukocyte antigen B27 (HLA-B27). Although the pathogenesis of SpA is multifactorial and only partially understood, HLA-B27 has consistently been identified as the major genetic risk factor, particularly in axial SpA, where up to 90% of patients with AS are HLA-B27 positive. However, the prevalence of HLA-B27 varies considerably among SpA subtypes and across different ethnic populations, and its presence is neither necessary nor sufficient for disease development. These observations highlight the complex genetic architecture underlying SpA. In addition to HLA-B27, numerous other HLA and non-HLA susceptibility loci involved in immune regulation, inflammation, and host–microbe interactions have been implicated in disease pathogenesis. Nevertheless, the precise mechanisms through which these genetic factors contribute to disease initiation, progression, and clinical heterogeneity remain incompletely defined, warranting further investigations to refine our understanding of SpA biology and identify novel therapeutic targets.

This also means that for a multi-faceted disease like SpA, diagnosis and treatment are often troubling even for experts. This problem stems from a lack of understanding of the underlying fundamental physiological and genetic pathogenesis mechanisms, and consequently a need for more accurate and precise classifications of the various subtypes. They have also been historically referred to as “seronegative spondyloarthropathies” due to the absence of rheumatoid factor—a protein made by the immune system to attack healthy tissues. This further obfuscates diagnosis when considering the possibility of other diseases such as seronegative rheumatoid arthritis [2].

Currently, the standard procedure of diagnosis looks at plain films of sacroiliac joints and spines, inflammatory markers and blood tests, MRI scans, radiographs, physical features of inflammation, etc. There is no single deciding diagnostic criterion, with delays of up to 6–8 years due to several factors including but not limited to a lack of awareness of this rare disease and poor extra-articular manifestations (EAMs) resulting in difficulties in confirming the exact subtype [3]. Regarding treatment, varying degrees of success have been attributed to anti-inflammatory drugs, antibodies, and other medications. In successful treatments, patients often experience substantial relief of pain and stiffness, but remissions are typically short. Additionally, “success” depends on the specific type of SpA as the root causes and pathways are largely different. In less successful cases, complications can reduce efficacy and demand constant trial-and-error as therapy is also dependent on the patient’s pharmacogenetic response. Factors such as sex and ethnicity have also been found to correlate with differences in both SpA progression, severity and drug response [4]. As a result of inconsistent symptom manifestations and the complexity of the disease, what is considered the right, long-term therapy can be unclear to this date.

This mini-review aims to briefly compile the latest findings in the research field of SpA, focusing on its pathogenesis, hypothesized mechanisms of action and current and emerging antibody discovery platforms. We hope to summarize current gaps and challenges in SpA research and offer a perspective on how heavy computational methods such as in silico predictions and simulations, large language models and multi-omics technologies can become indispensable tools of SpA antibody discovery, diagnostics and mechanism elucidation.

## 2. SpA Pathogenesis and Therapeutics Landscape

### 2.1. SpA Pathogenesis

Generally, SpA arises as a result of excessive inflammation around the spine or peripheral joints, culminating in abnormal bone formation. Several postulated hypotheses of the disease mechanism include the arthritogenic peptide hypothesis, HLA-B27 misfolding hypothesis, HLA-B27 homodimer formation hypothesis, endoplasmic reticulum aminopeptidase (ERAP) polymorphism hypothesis and gut inflammation and dysbiosis hypothesis [5]. While the exact mechanistic pathways have not been elucidated, there is increasing evidence of gut dysbiosis—intestinal inflammation and homeostatic imbalance due to impaired gut barriers and microbiome changes—playing a significant role in the pathogenesis of SpA. This, however, does not invalidate the other hypotheses, especially given that SpA is extremely heterogeneous with a wide range of subtypes. In the early stages of SpA, inflammation and pain typically start from the lower back and buttocks, though they can also present initially in the peripheral joints. As SpA progresses, EAMs may start to develop, leading to uveitis (eye inflammation), enthesitis (enthesis inflammation), and dactylitis (finger or toe inflammation). A crucial point in the disease progression is when excessive inflammatory signals released by γδ T cells, mucosal-associated invariant T (MAIT) cells and Th17 cells activate osteoclasts, causing osteoproliferation and bone erosion. The body thus attempts to repair the erosion, albeit leading to excessive and abnormal bone formation that is irreversible structural damage. Ectopic chondroproliferation (syndesmophytes) develops and fuses joints in the spine. This then develops into ankylosis in the more severe AS condition in late SpA.

The interleukin-17/interleukin-23 (IL-17/IL-23) interaction is known as one of the most significant contributors to SpA immunopathogenesis. IL-23 promotes the expansion and survival of IL-17-producing cells such as Th17, γδ T cells and innate lymphoid cells (ILCs), while IL-17 stimulates the production of pro-inflammatory cytokines, chemokines and matrix metalloproteinases (MMPs), thereby amplifying inflammation and tissue remodelling. Elevated levels of IL-17/23 have been consistently observed in SpA patients. Despite the immunological role of IL-23, many clinical trials have reported failures in IL-23 inhibitors [6], causing most therapeutics to switch to inhibiting the more downstream regulator IL-17. The mystery of the ineffectiveness of IL-23 inhibitors remains unresolved and is one of the reasons why we need more scientific experiments and hypotheses to clarify the pathogenetic mechanisms in SpA.

Tumor necrosis factor alpha (TNF-α) is also a well-known cytokine that promotes leukocyte recruitment, osteoclastogenesis and inflammatory signaling pathways through binding to its target receptors TNFR1 and TNFR2. As a molecule produced at elevated levels in diseased individuals, it is an important target similar to IL-17/IL-23 in order to neutralize excessive inflammation, potential bone erosion and aberrant permanent bone formation in the later stages of SpA.

**Figure 2:**
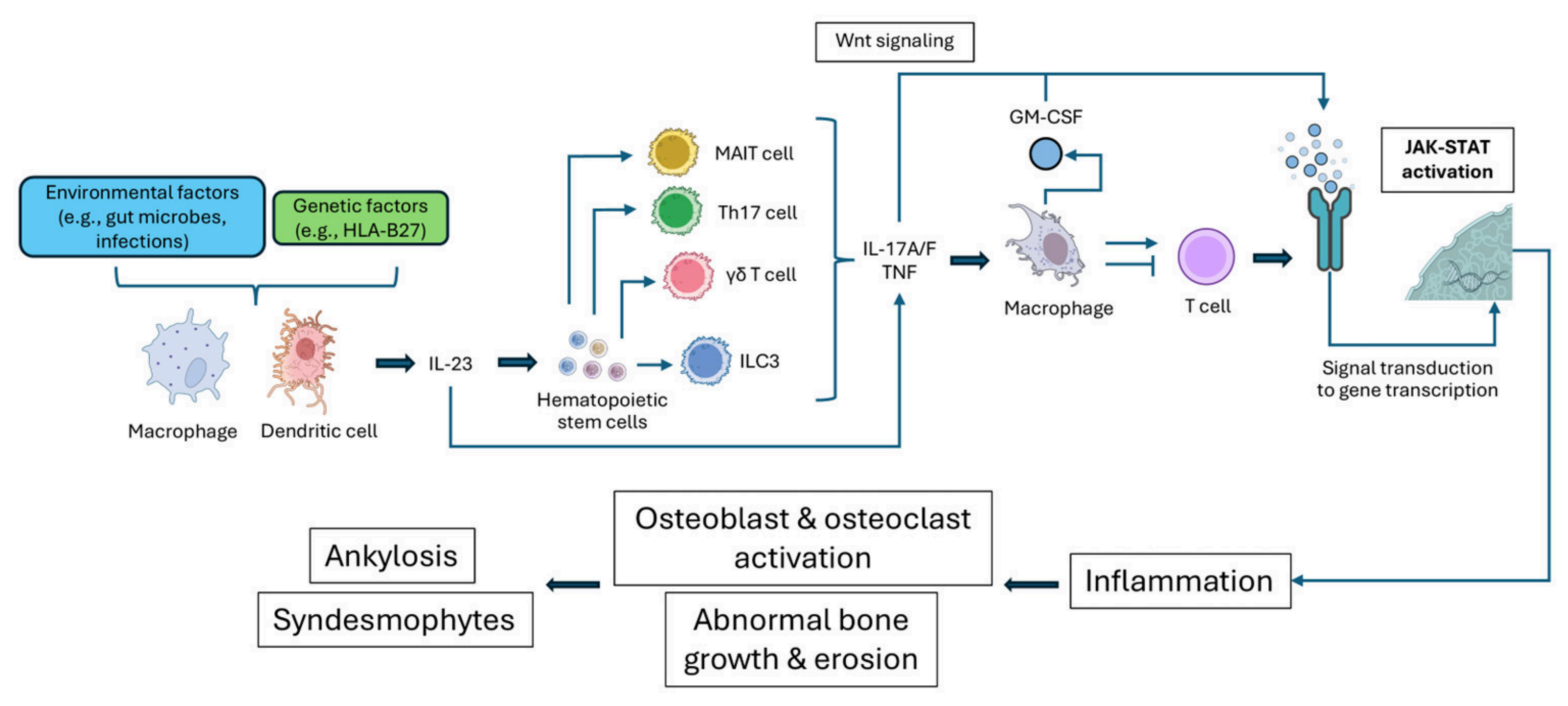
A simplified overview of the inflammatory pathways in SpA disease development involving key molecules and cells. Both genetic and environmental factors are associated with SpA disease where HLA-B27 and gut microbe dysbiosis are thought to activate immune cells such as macrophages and dendritic cells. This process activates the downstream signaling pathways involving IL-23, generating tissue-resident cells such as MAIT, Th17, γδ and ILC3 cells that produce IL-17 and TNF. These two key proteins cause subsequent canonical and non-canonical Wnt and JAK-STAT activation, inflammation, abnormal bone formation and thus ankylosis and peripheral manifestations. SpA, spondyloarthritis; MAIT, mucosal-associated invariant T; Th17, T helper 17; ILC3, type 3 innate lymphoid cell; GM-CSF, granulocyte macrophage-colony stimulating factor. Illustrations used were taken from NIAID NIH BioArt Source [8,9,10,11,12,13,14,15,16,17].

Recent research has also revealed more findings on various key molecules in bone remodeling involving bone morphogenetic proteins (BMPs), Wnt signaling pathways (both canonical and non-canonical) and osteoblast and osteoclast activation, although more still needs to be done to build a concrete picture of the molecular interactions [7]. Chronic inflammation may paradoxically stimulate osteoproliferation after initial tissue injury, indicating that effective suppression of inflammation does not always completely prevent structural progression. A simplified diagram illustrating the inflammatory pathways as well as key molecules and cells in SpA disease development is in Figure 2 below.

### 2.2. Gut-Joint Axis

The latest research findings have suggested the gut-joint axis as an important contributor to SpA immunopathogenesis. Subclinical intestinal inflammation has been associated with axial SpA, and patients with IBD frequently develop SpA manifestations. Many review articles have proposed that alterations in gut microbiota composition, or dysbiosis, can activate mucosal immunity and promote systemic inflammation through increased intestinal permeability and cytokine production, leading to the development of SpA [18,19,20,21]. These studies revealed differences in the intestinal microbiota environment in SpA patients compared to healthy individuals, suggesting that gut-microbe interactions are at play.

Gracey et al. (2020) cautions that while gut and joint inflammation in SpA are linked, their causative relationship is still elusive [22]. The hypothesis that inflammatory events in the gut precede and directly lead to inflammatory events in the joint has been challenged due to five reasons: first, SpA is still able to develop in the absence of gut inflammation; second, many genetic risk factors associated with SpA have unknown function in arthritis; third, knowledge of the biology underpinning the role and extent of type 3 immune cytokines in both gut and joint is lacking; fourth, gut dysbiosis has not been confirmed to have causative effects on gut inflammation; lastly, whether the failure of current therapies for SpA is related to gut inflammation in unknown.

As of 2025, researchers have discovered potential mechanisms in which microbes may cause SpA, such as molecular mimicry, microbial translation, dysregulation of mucosal immunity, production of immunomodulatory metabolites and immune cell trafficking. Methods developed to make such discoveries possible include IgA-sequencing, anti-bacteria IgG antibodies and identifying microorganism-sensitive T cells using defined bacteria to stimulate T cells and measuring activation-induced markers on them. However, many confounding factors such as disease features, type of treatments, comorbidities and study population are limiting advances in SpA [20].

Together, the pathogenic mechanisms described above highlight the complex interplay between immune dysregulation, tissue-specific inflammation, and the gut–joint axis in SpA. Increasing understanding of these disease mechanisms has provided a rationale for targeting key inflammatory pathways therapeutically. This has led to the development of several effective biologic therapies and continues to drive the emergence of new antibody-based therapeutic strategies for SpA, as discussed in the following section.

### 2.3. Current and Emerging Antibody Therapies for SpA

Pharmacologically, NSAIDs are the first choice to combat inflammation, prevent worsening symptoms and relieve pain. These include naproxen and celecoxib. However, they are often not ideal therapies and are unable to resolve the root cause of the disease. Therefore, depending on the diagnosed type of SpA disease, patients are then prescribed biologic/targeted synthetic/conventional synthetic disease-modifying anti-rheumatic drugs (b/ts/csDMARDs) which function as TNF, IL or Janus kinase (JAK) inhibitors. JAK inhibitors such as tofacitinib, upadacitinib and filgotinib have been demonstrated to be effective alternatives to bDMARD-naïve patients. A summarized list of the current therapies used to treat SpA and those under research can be found in Table 1 below.

IL-17/23 inhibitors inhibit IL-17A and IL-23 signalling pathways respectively, thereby reducing inflammatory signalling and tissue damage. Targeted therapy TNFi neutralises soluble and membrane-bound TNF-α, which suppresses downstream inflammatory signalling by reducing leukocyte migration, cytokine production and osteoclast activation. Adalimumab, infliximab, etanercept, golimumab and certolizumab pegol are the most commonly used and researched especially for axSpA. However, there is still a substantial lack of efficacy where approximately 30–40% of patients still exhibit inadequate response or adverse drug reactions to TNF inhibitors [52]. Many patients also have contraindications when taking these therapeutics, resulting in the need for both more effective and personalized treatment methods. Combined with the fact that there is still no cure for SpA, novel drug development is still an ongoing and tectonic process to find truly effective drugs in treating different facets of SpA.

The emerging antibody therapies which have been under research for the past few years include the following: nanobodies (sonelokimab, IL-17 inhibitor) for PsA [53], IL-17A/F dual inhibitors such as ORKA-002 for PsA [54], affibodies (izokibep, IL-17 inhibitor) for PsA [55], T-cell bispecific antibodies (BsAbs) for axSpA [56], tulisokibart MK-7240 [57], aptamers targeting IL-17 [58] and a new IL-17 inhibitor xeligekimab. Xeligekimab is a fully human monoclonal IgG4 antibody targeting IL-17A developed in China, demonstrating significant efficacy and a favorable safety profile in patients with active AS, reductions in disease activity (ASDAS-ESR/CRP), improved function (BASFI), and spinal mobility (BASMI) [59]. Nanobodies (Nbs) are single-domain antibodies derived from camelid heavy-chain antibodies where their small size enhances tissue penetration and stability. Affibody molecules are small and robust protein with a high affinity towards a specific protein target, with its original protein scaffold based on the IgG binding domain of Staphylococcal protein A. An affibody consists of three alpha helices with 58 amino acids and a small approximate mass of 6.5 kDa [60], and can be customized to a specific target desired through mutation of the 13 amino acids in the first two helices. Although first described long ago in 1997 [61], they have recently surfaced as potential therapeutics for a variety of diseases including SpA. Other potential therapies which are still undergoing clinical trials include granulocyte-macrophage colony-stimulating factor (GM-CSF) inhibitors, mitogen activated protein kinase-activated protein kinase-2 (MK2 inhibitor), T-cell directed immunotherapy, macrophage inflammatory protein (MIP), and mechanistic target of rapamycin (mTOR) [62]. These should be kept in view as candidate therapeutics for treating the various SpA subtypes.

There are benefits and limitations to these new therapeutic strategies. A summary of the newest therapeutics and their pros and cons is listed in Table 2 [63,64,65,66]. At this point in time, their effectiveness is still undergoing research; thus, it is difficult to make any meaningful conclusions. However, we speculate that the nanobodies and affibodies can be incorporated into a new treatment method that allows the molecule to reach its target more precisely given their higher stability and smaller mass. While they may have a short half-life, and nanobodies have low serum persistence and lack an Fc region, such limitations can be overcome through modifications of the main body. One example of this is the designed inclusion of an albumin-binding domain in sonelokimab to improve its half-life, thereby increasing its target selection and accumulation in tissues [53].

## 3. Current Methods and Advances in Antibody Discovery Platforms

### 3.1. Current Antibody Discovery Platforms

Early monoclonal antibody (mAb) therapies used hybridoma technology which involved fusing antibody-producing B cells with immortalized myeloma cells. It is a labor-intensive yet cost-effective in vivo method of generating mAbs with the highest antigen affinity. However, such hybridoma-derived antibodies often exhibited immunogenicity due to murine protein sequences. Consequently, humanization techniques were developed to create humanized transgenic mice containing human antibody gene sequences, allowing for the development of fully human monoclonal antibodies with much lower immunogenicity [67]. While hybridoma technology has dramatically improved and is still used today, phage display technology has emerged as a superior high-throughput in vitro method where bacteriophages express antibody fragments on their surface, allowing scientists to identify antibodies with strong binding properties against specific targets at high affinity. Other related techniques such as yeast/mammalian cell display, ribosome display and single B-cell cloning perform similar functions but with different molecular tools (Table 3) [68,69,70].

Phage display has been a pivotal driver of antibody discoveries, such as adalimumab whose process began in 1993 and approval came into place nine years later. Transgenic mouse technology has also produced many drugs such as golimumab. According to McCafferty (2010), the identification of new validated targets would be the bottleneck in drug discovery and development [71]. This is largely due to the financial and time cost incurred. While this bottleneck has been diminished, there remain two critical breakpoints in order to move the field forward: first, better clinical trials in terms of efficiency and patient recruitment need to be designed; second, our fundamental knowledge of human biology, disease mechanisms as well as model systems.

The current state of the field is moving toward the integration of multi-omics technologies, long-read sequencing and various in silico discovery methods to identify possible biomarkers and therapeutic options in a more efficient and accurate manner. We foresee that once biomarkers can be reliably predictive, precision/personalized medicine has huge potential in enabling individualized treatment selection and reducing unnecessary and costly exposure to ineffective therapies.

### 3.2. Advances in Antibody Discovery Platforms

The advancement of NGS technology, high-throughput data analysis and development of large supercomputing clusters have made antibody discovery more feasible, though not completely ready, without relying on wet-lab experiments. Machine learning (ML) and artificial intelligence (AI) have been transforming therapeutic antibody design through enabling antibody-antigen interaction predictions, binding affinity optimizations and structural modelling-designed bispecific antibodies. For instance, bimekizumab is a monoclonal bispecific antibody that has been computationally designed and FDA-approved to better treat psoriasis, PsA, AS and nr-axSpA. A review by Matsunaga and Tsumoto [70] compiled a list of publications focusing on antibody design, discovery and optimisation using ML approaches such as one-hot encoding, bidirectional encoder representations from transformers (BERT) models and a modified version RoBERTa, and long short-term memory (LSTM) models. Many of these are large language models (LLMs) which are pre-trained on a huge amount of data obtained from display methods and antibody screening methods.

Fundamentally, they work by using neural networks consisting of encoders and decoders that extract and understand relationships from protein sequences and structures [70]. By performing computational and statistical methods (e.g., calculating joint probability distributions, tokenization) on sequence data of candidate antibodies, whether from pre-existing experimental results or public databases, an ML model can be trained to identify patterns in sequence-structure-function relationships and accurately predict properties of novel antibody sequences such as binding affinity, expression yield, and stability for novel candidates. In one of the latest protein design works, the evolutionary scale modeling (ESM3) model, a deep learning (DL) model, was used to generate a distant functional green fluorescent protein (GFP) that could have possibly evolved naturally within 500 million years, which they termed esmGFP [72]. Thus, ML and DL models can bear the grunt of doing repetitive, complex and time-costly computations to come up with novel antibodies with the predicted desired therapeutic properties even for SpA.

In another example to demonstrate this computational approach in an autoimmune disease rheumatoid arthritis, Barkhordari et al. (2020) [73] integrated homology modeling with molecular dynamics simulations to engineer novel bispecific antibody fragments neutralizing TNF-α and IL-23 as a potential drug candidate. This in silico study is a proof-of-concept of how computer-aided drug design can be used in antibody discovery for SpA as well [73].

Chen et al. (2024) detailed the latest ongoing studies on ML-assisted research on SpA, stating that ML is an emerging central focus of research across multiple disciplines [74]. Their article highlighted that there has been much research incorporating ML conducted on five key areas: early diagnosis, stratification, disease monitoring, drug efficacy assessment and comorbidity prediction.

In early diagnosis, ML methods such as linear and logistic regressions, least absolute shrinkage and selection operator (LASSO), support vector machine (SVM), random forest (RF), nomograms, weighted correlation network analysis (WGCNA) and minimum-Redundancy-Maximum-Relevance (mRMR) were used in the integration of clinical and radiological data (medical claims, electronic health records, sacroiliac MRI radiomics, etc.) to construct models for improving diagnosis of axSpA with a high AUC statistic of up to 0.93; in stratification, 3D convolutional neural networks (CNNs), k-nearest neighbours (kNN) and deep learning (DL) were used on CT and MRI image scans to distinguish AS, nr-axSpA and pSpA, with the prediction models reportedly outperforming radiologists with 9 years of experience; in disease monitoring, generalized linear models (GLMs), artificial neural networks (ANNs) and multiclass Bayesian networks were used alongside modified stoke ankylosing spondylitis spinal scores (mSASSS), NSAID and TNF drug usage, as well as wearable trackers to predict spine structural damage and AS recurrence; in drug efficacy assessment, ANN was determined to be the best model amongst logistic regression, SVM, RF and XGBoost (AUC: 0.783, 0.719, 0.699, 0.761, 0.713) in predicting the timing of drug intervention and efficacy, particularly TNF; in comorbidity prediction, LASSO, RF, XGBoost and SVM models were used to establish diagnostic models which found five genes (TNF, CCL3, PIK3CG, PTGER2 and IFNAR1) that could be used as potential biomarkers to predict low bone density (LBMD) risk in AS patients, who often suffer from LBMD, osteoporosis and vertebral fractures due to inflammation causing bone erosion. To predict cardiovascular disease in AS patients, SVM, RF and kNN (AUC: 0.7, 0.73, 0.64) were applied to clinical data such as sex, age, blood pressure, CRP and ESR, as well as radiological data, identifying CRP as the most crucial predictor.

While these computational and statistical tools are relatively successful with the lowest AUC at 0.64 and highest at 0.93, they are still immature and can be further refined to become promising tools for more efficient discovery of novel therapeutics for SpA as well as clinical use. More scientists should seriously consider in silico antibody discovery tools and models and critically evaluate their effectiveness in improving SpA’s drug treatment options, especially when considering personalized treatment for individual patients. Although these tools are computationally successful, they have not yet actually advanced our biological knowledge of the foundational mechanisms in SpA pathogenesis, so there is still more relevant work to be fulfilled in this area.

## 4. Challenges in Antibody Discovery, Diagnostics and Prognostics for SpA

### 4.1. Target and Therapeutic Discovery Challenges

Therapeutic antibody discovery for SpA is complicated by the disease’s heterogeneity, encompassing multiple phenotypes, including axSpA, PsA, RA and EA [75]. This heterogeneity is driven by diverse genetic susceptibilities, tissue-specific immune-cell populations, and complex cytokine signaling networks, resulting in substantial variability in disease mechanisms and therapeutic responses. Consequently, identifying universal therapeutic targets remains challenging, as inhibition of a single inflammatory pathway frequently triggers compensatory activation of alternative cytokine networks, contributing to complications of both primary and secondary treatment [76,77].

Although biologic therapies targeting TNF-α and the IL-17 pathway have transformed the management of SpA, a substantial proportion of patients have inadequate responses or lose efficacy over time. Furthermore, tissue-specific disease mechanisms complicate the selection of therapeutic targets. A notable example is the IL-23 pathway. Inhibition of IL-23 demonstrates clinical efficacy in psoriasis and peripheral manifestations of PsA, but has consistently failed to improve outcomes in established axSpA. In a phase II trial of 159 patients with active AS, risankizumab, an IL-23 inhibitor, did not significantly improve the primary ASAS40 endpoint as compared to the placebo group at week 12 (15.0–25.5% vs. 17.5%, respectively) [78], despite targeting the same pathway that is therapeutically effective in other SpA-related conditions [79,80]. Similarly, three randomized trials of ustekinumab in axSpA failed to demonstrate clinically meaningful improvement over placebo [81]. These findings highlight important differences in disease biology across SpA domains and emphasize the need for more precise target selection in antibody development.

Another major challenge is the dissociation between inflammation and pathological new bone formation. While current biologic therapies do effectively suppress inflammatory activity, they are not consistent in preventing the formation of syndesmophytes, ankylosis, and irreversible structural damage, which remain the principal causes of long-term disability in patients with SpA [82,83].

In addition, pre-clinical validation remains a major obstacle because existing animal models fail to fully recapitulate the complex, multisystem pathology of human SpA. For example, HLA-B27 transgenic and SKG models reproduce key inflammatory and entheseal features, whereas other models better capture pathological bone formation; however, most fail to simultaneously reproduce the full spectrum of axial disease, new bone formation as well as extra-articular manifestations seen in humans. These limitations may reduce the predictive value of preclinical efficacy and safety studies and highlight the need for complementary or more translational models [84]. Furthermore, biologic drug development is associated with substantial technical complexity, high development costs, and the risk of immunogenicity, in which patients develop anti-drug antibodies that reduce therapeutic efficacy or accelerate drug clearance [85]. Collectively, these challenges contribute to high attrition rates during antibody development and delay the translation of promising therapeutic candidates into clinical practice.

### 4.2. Diagnostic and Prognostic Biomarker Challenges

In parallel with discovery hurdles, the clinical implementation of precision medicine in SpA remains limited by the lack of reliable diagnostic and prognostic biomarkers to guide individualized treatment strategies. Current routine treatment decisions rely primarily on clinical manifestations, imaging findings, and conventional inflammatory markers such as CRP and ESR, which have limited sensitivity and specificity and do not consistently reflect disease activity or predict structural progression [79,86].

Advances in high-throughput technologies, including genomics, epigenomics, transcriptomics, proteomics, metabolomics, and single-cell RNA sequencing, have identified numerous candidate biomarkers associated with immune dysregulation, cytokine signaling, and tissue remodeling. These molecular profiling approaches have improved understanding of disease heterogeneity and revealed distinct inflammatory endotypes that may facilitate patient stratification and support the development of targeted antibody therapies. Nonetheless, most proposed biomarkers remain confined to research settings due to limited validation, poor reproducibility across patient cohorts, and a lack of standardized assays for routine clinical use [86,87,88].

In addition to diagnostic limitations, the lack of predictive biomarkers remains a major obstacle to optimizing biologic therapy. Several circulating inflammatory mediators, including IL-17A, TNF-α and MIF, as well as extracellular matrix turnover markers such as C2M and C3M, have been investigated as potential biomarkers of disease activity and structural changes [79,86,89]. However, their ability to reliably predict treatment response or disease progression remains limited, and none has yet demonstrated sufficient clinical utility for routine patient stratification [79,86,89]. Furthermore, no validated biomarker has been established to accurately predict therapeutic response to specific biologic agents or the risk of anti-drug antibody development, resulting in a largely empirical, trial-and-error approach to treatment selection [79,90]. The identification and validation of robust molecular biomarkers therefore remain critical to enabling precision medicine, improving patient stratification and optimizing the efficacy as well as long-term outcomes of antibody-based therapies in SpA.

## 5. Future Directions in Precision Antibody Discovery and Clinical Translation

Recent advances in AI and DL are transforming antibody discovery by enabling prediction of antibody-antigen interactions, antibody structure, affinity, and developability, thereby improving the efficiency of therapeutic antibody development [91,92,93]. When integrated with multi-omics and high-throughput experimental platforms, such as deep mutational scanning (DMS), these approaches could facilitate rational antibody design, affinity maturation, and prioritization of promising candidates while reducing the experimental search space [94,95]. In SpA, integrating multi-omics data with AI-based approaches could facilitate the discovery of disease-relevant targets and biologically distinct patient subgroups, paving the way for targeted antibody therapies [96,97]. However, robust experimental validation remains essential before these computational predictions can be translated into clinical practice.

Future advances in understanding SpA pathogenesis are expected to drive a transition from conventional “one-size-fits-all” treatment approaches to precision antibody therapeutics tailored to individual disease mechanisms and patient-specific molecular profiles. Integrating high-throughput multi-omics technologies, such as genomics, transcriptomics, proteomics, metabolomics, single-cell RNA sequencing, and spatial transcriptomics, offers an opportunity to define the molecular heterogeneity and disease-specific endotypes of spondyloarthritis (SpA). These technologies can help identify potential therapeutic targets. In the future, combining these datasets with bioinformatics and AI may enable systematic prioritization of disease-relevant targets, prediction of patient-specific therapeutic responses, and identification of molecular biomarkers for patient stratification. Additionally, integrating AI-driven antibody design with high-throughput experimental validation could expedite the transition from target discovery to the development of optimized therapeutic antibodies. This approach supports a more mechanism-based strategy for precision therapy in SpA. Similarly, transforming these strategies into clinical practice will require prospective validation and integration with broader clinical datasets to confirm their utility.

**Figure 3:**
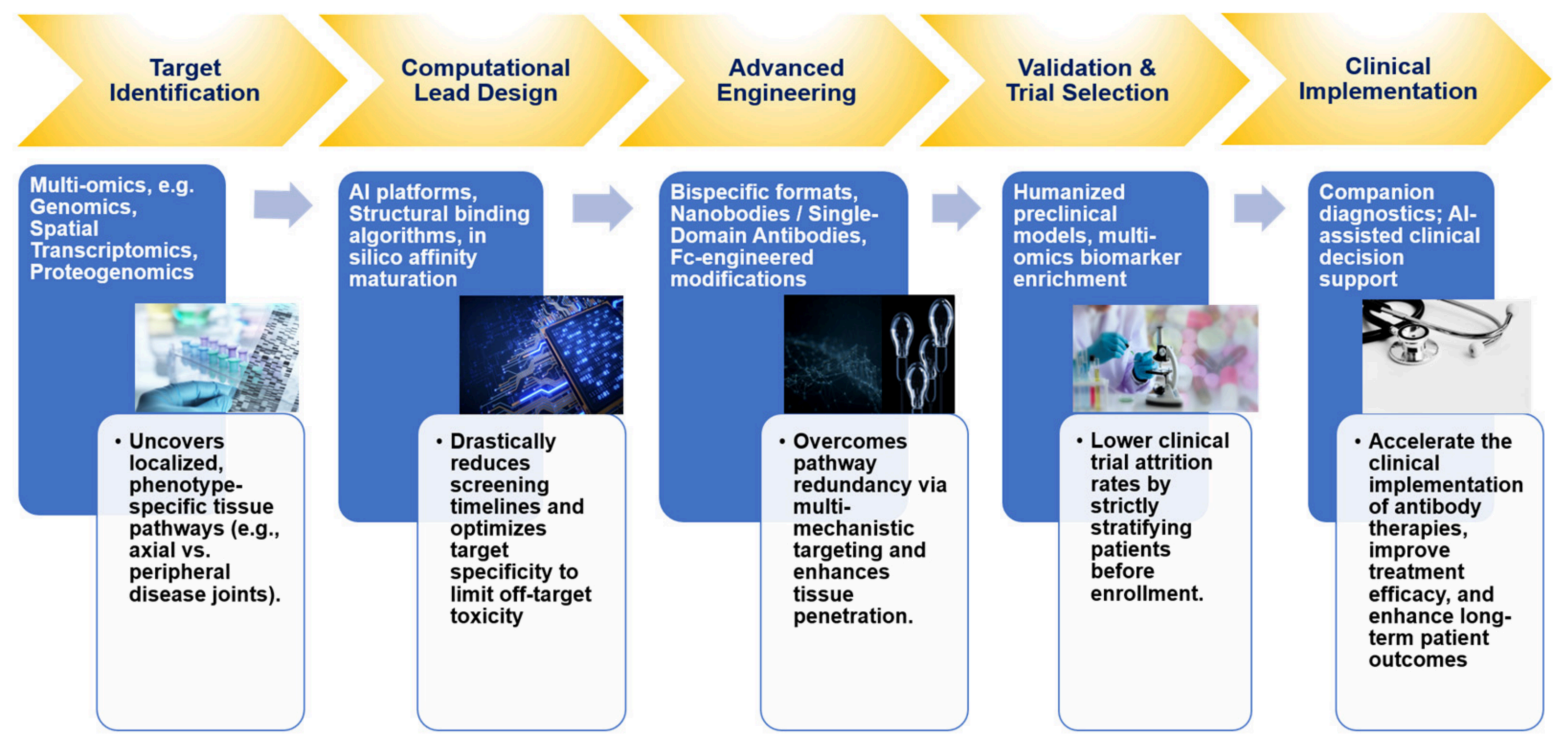
Future Directions in Precision Antibody Discovery Pipeline. A workflow on future precision antibody discovery. Yellow boxes on top: stages; blue boxes in the middle: core technologies involved; white boxes at the bottom: strategic impact on SpA management.

Discovered targets are subsequently translated into next-generation therapeutic modalities, including bispecific and multispecific antibodies, highly tissue-permeable nanobodies and strategically engineered Fc-based molecules, before undergoing validation in humanized preclinical models and biomarker-enriched clinical cohorts. Concurrently, advances in antibody engineering are expanding therapeutic possibilities by enabling the simultaneous modulation of multiple inflammatory pathways, thereby overcoming cytokine redundancy, enhancing therapeutic efficacy, and potentially reducing treatment resistance. The future precision antibody discovery pipeline, from target identification to clinical implementation, is summarized in Figure 3.

The successful clinical translation of next-generation antibody therapeutics will depend on integrating biomarker-guided precision medicine throughout the drug development pipeline. Future clinical trials are expected to incorporate molecular biomarkers for patient stratification, prediction of therapeutic response, and monitoring of immunogenicity, thereby enabling more efficient trial designs and improving clinical outcomes [88]. In parallel, emerging preclinical platforms—including humanized animal models, patient-derived organoids, and organ-on-chip technologies—are expected to improve predictions of efficacy and safety, thereby reducing the high attrition rates associated with biologic drug development.

Looking ahead, the realization of precision medicine in SpA will require the systematic integration of longitudinal patient datasets with multi-omics information, including genomic variants, transcriptomic signatures, proteomic profiles, gut microbiome composition, and real-world clinical data. Combined with advances in AI and ML, these comprehensive datasets will assist in facilitating the identification of molecular endotypes, enable dynamic prediction of disease progression and therapeutic response, and support continuous refinement of individualized treatment strategies. Collectively, these innovations are expected to accelerate the development of personalized antibody therapeutics that maximize clinical efficacy, minimize adverse effects, and improve long-term outcomes for patients with SpA.

## 6. Conclusions

SpA has had important advances in the understanding of its biology, diagnostics, prognostics and therapeutics since the 2000s. But such advances are still insufficient for scientists and clinicians to find truly effective therapies. Future advances in antibody therapeutics for SpA should and will rely on integrating multi-omics technologies, computational- or AI-driven approaches, biomarker discovery, and a deeper understanding of disease pathogenesis to identify molecular endotypes and enable precision treatment strategies. Emerging approaches, including bispecific antibodies, engineered antibody formats, and combination therapies, hold considerable promise for overcoming cytokine redundancy, improving therapeutic efficacy, and minimizing treatment resistance. Continued collaboration among basic scientists, clinicians, and the pharmaceutical industry will be essential to translate these advances into safer, more effective, and personalized antibody-based therapies, ultimately improving long-term clinical outcomes and quality of life for patients with SpA.

## Figures and Tables

**Table 1:** A table of the four main therapeutic drug classes mainly used to treat SpA, commonly used drugs within each class, their subtype (mechanism of action) and efficacy. These drugs have been approved for therapeutic use in SpA following clinical trials.

Class of Drug	Drug	Subtype	Disease
NSAIDs	Naproxen	Non-selective	SpA [23]
	Celecoxib	COX-2 inhibitor	axSpA [24]
bDMARDs	Adalimumab	TNF-α inhibitor	axSpA [25]; JIA [26]
	Infliximab	TNF-α inhibitor	axSpA [27]; PsA [28]
	Golimumab	TNF-α inhibitor	axSpA [29,30]; PsA [31,32]
	Certolizumab pegol	TNF-α inhibitor	AS [33]; nr-axSpA [34]; PsA [35]
	Bimekizumab	IL-17A/F inhibitor	axSpA; PsA [36]
	Secukinumab	IL-17A inhibitor	axSpA [37,38]; PsA [39]
	Ixekizumab	IL-17A inhibitor	AS [40]; nr-axSpA [41]; PsA [42]
tsDMARDs	Upadacitinib	JAK inhibitor	AS [43]; PsA [44]; IBD-SpA * [45]
	Tofacitinib	JAK inhibitor	axSpA [46]
	Filgotinib *	JAK inhibitor	AS [47]; PsA [48]
	Etanercept	TNF-α inhibitor	axSpA * [49]; pSpA [50]
csDMARDs	Methotrexate	NF-kb inhibitor	axSpA * [51]; pSpA [50]
	Sulfasalazine	NF-kb inhibitor	SpA [23]

* Not widely approved, limited evidence.

**Table 2:** A table summarizing the pros and cons of the newest therapeutic formats in treating SpA.

Therapeutic	Description	Advantages	Disadvantages
Nanobodies	Small single-domain antibodies (12–15 kDa) derived from camelid heavy-chain antibodies	Able to bind to concave sections of antigens that are inaccessible to mAbs Greater tissue penetration High thermal stability and pH and protease resistance Low to no immunogenicity thus having low risk of ADR Easier multimerization (multivalency/multiparatopicity/multispecificity) Relatively simpler to modify, produce and purify	Their monovalent format often requires modification, i.e., multivalency, for them to display sufficient therapeutic as well as diagnostic capacity, thereby often adding an additional complex step Low serum persistence or rapid renal clearance as the threshold for glomerular filtration is 50–60 kDa, thus presenting a disadvantage in diagnostic screening and therapeutic applications Nbs lack an Fc region and therefore cannot exert effector functions associated with specific moieties Short half-life
Bispecific antibodies	Artificial antibodies that can simultaneously bind to two distinct antigens or epitopes on the same antigen	Lack of an Fc region simplifies the structure and reduces production costs while maintaining high yields Modular nature of this approach allows for the customization of valency and specificity, supporting diverse therapeutic applications BsAb binding of two targets on the same cell increases drug specificity and reduces off-target side effects	Lack of an Fc region means BsAbs do not benefit from the neonatal Fc receptor’s protective mechanisms, resulting in a shorter circulation time in the body Difficult to optimize target antigen selection to achieve the desired synergistically therapeutic effect Higher risk of anti-drug antibodies Complex dosing and clinical trial design
Affibodies	Small proteins (~6.5 kDa) lacking in disulfide bridges engineered to bind to a large number of target proteins or peptides with high affinity and specificity	Simpler and smaller than Abs Able to sustain a wide range of extreme variations in pH and temperature	Short biological half-life

**Table 3:** A table summary of the current methods for antibody discovery and library screening adapted from Matsunaga & Tsumoto [70].

Method	Description	Advantages	Disadvantages
Hybridoma	B cells from immunized (transgenic) mice are fused with immortalized myeloma cells and screened.	Gold standard for reproducing mAbs Cheaper than below methods	Slow timeline Low throughput
Phage/Yeast/Mammalian display	Antibody fragments are displayed on phage coat proteins/yeast cell surface/mammalian cell surface. Libraries are screened against an antigen via panning or FACS.	More high throughput than hybridoma with better selection and large library sizes (<10^11^) Faster than hybridoma	Requires specialized equipment or cell lines Amplification bias during panning process
Ribosome/cDNA display	Antibody-mRNA complexes are formed and stabilized on ribosomes, allowing for cell-free selection based on antibody-antigen binding.	High throughput with larger library sizes (<10^15^) More suitable for toxic and difficult antigens	mRNA-ribosome-protein complexes can be unstable and prone to dissociation during selection. mRNA may be degraded by nucleases in the reaction mixture Faster than hybridoma and display methods
Single B-cell cloning	Single B cells specific to target antigen are isolated from organism, sequenced via RT-PCR or scRNA and specific antibodies are harnessed.	High throughput and direct recovery of human antibodies with naturally paired chains without animal immunization Faster than hybridoma and display methods	Expensive compared to above methods Dependent on prior knowledge of target of interest

## Data Availability

No new data were created or analyzed in this study. Data sharing is not applicable to this article.

## Abbreviations

The following abbreviations are used in this manuscript:

AI	Artificial intelligence
ANN	Artificial neural network
AS	Ankylosing spondylitis
ASDAS	Ankylosing spondylitis disease activity score
AUC	Area under curve
BASFI	Bath ankylosing spondylitis functional index
BASMI	Bath ankylosing spondylitis metrology index
BERT	Bidirectional encoder representations from transformers
BMP	Bone morphogenetic protein
BsAb	Bispecific antibody
CNN	Convolutional neural network
CRP	C-reactive protein
DL	Deep learning
DMARD	Disease-modifying anti-rheumatic drug
EA	Enteropathic arthritis
EAM	Extra-articular manifestations
ESM	Evolutionary scale modelling
ESR	Erythrocyte sedimentation rate
GM-CSF	Granulocyte macrophage-colony stimulating factor
HLA	Human leukocyte antigen
IBD	Inflammatory bowel disease
IL	Interleukin
JAK	Janus kinase
JIA	Juvenile idiopathic arthritis
kNN	k-nearest neighbours
LASSO	Least absolute shrinkage and selection operator
LBMD	Low bone density
LLM	Large language model
LSTM	Long short-term memory
MAIT	Mucosal-associated invariant T
MIF	Macrophage migration inhibitory factor
MIP	Macrophage inflammatory protein
MK2	Mitogen-activated protein kinase activated protein kinase 2
MMP	Matrix metalloproteinase
mAb	Monoclonal antibody
mRMR	Minimum-Redundancy-Maximum-Relevance
mSASSS	Modified stoke ankylosing spondylitis spinal scores
mTOR	Mechanistic/Mammalian target of rapamycin
NF-kB	Nuclear factor kappa-light-chain-enhancer of activated B cells
NGS	Next-generation sequencing
NSAID	Non-steroidal anti-inflammatory drug
Nb	Nanobody
PsA	Psoriatic arthritis
RA	Rheumatoid arthritis
RF	Random forest
SVM	Support vector machine
SpA	Spondyloarthritis
TNF	Tumour necrosis factor
uSpA	Undifferentiated spondyloarthritis
WGCNA	Weighted correlation network analysis
